# House dust mite‐induced asthma alters BBB integrity, increases amyloid beta accumulation, and alters brain cytokine levels in *App^NL‐G‐F^* mice

**DOI:** 10.1002/alz.091066

**Published:** 2025-01-03

**Authors:** Bijayani Sahu, Suba Nookala, Angela M Floden, Nilesh Ambhore, Sathish Venkatachalem, Colin K Combs

**Affiliations:** ^1^ University of North Dakota, Grand Forks, ND USA; ^2^ North Dakota State University, Fargo, ND USA

## Abstract

**Background:**

Alzheimer’s disease (AD) is an age‐related neurodegenerative disorder affecting nearly 50 million individuals worldwide. Besides aging, various comorbidities can increase the risk of AD, such as asthma. However, the molecular mechanism(s) underlying this asthma‐associated AD exacerbation is unknown. This study was designed to explore the effects of house dust mite‐induced asthma on AD‐related brain changes using the *App^NL‐G‐F^* transgenic mouse model.

**Method:**

Male and female C57BL/6 wild type and *App^NL‐G‐F^* mice (8‐9 months old) were exposed to either saline or house dust mite (dose: 833µg/kg in saline) every alternate day for 16 weeks. Mice were sacrificed at the end of the experiment and broncho‐alveolar lavage fluid (BALF), lungs, blood, and brains were collected. BALF was analyzed for immune cell markers, inflammatory mediators, and LDH activity. Lung sections were stained with Alcian blue and Masson’s Trichrome to examine mucus and collagen production, respectively. The serum was analyzed for cytokine levels. Brain sections were immunostained for Aβ, GFAP, and collagen‐4. Finally, frozen hippocampi and cortices were used to perform Aβ ELISAs and cytokine arrays, respectively.

**Result:**

Dust‐mite exposure increased inflammatory cells, cytokine levels, and LDH activity in the BALF and increased the mucus and collagen production in the lungs from both sexes and genotypes, suggesting induction of a severe asthma‐like condition. This correlated with increased levels of serum cytokines in all dust‐mite‐exposed groups. In agreement with this peripheral change, hippocampi from asthma‐induced male and female *App^NL‐G‐F^* mice demonstrated elevated Aβ plaque load and increased soluble Aβ 1‐40/42 and insoluble Aβ 1‐40 levels. Dust‐mite exposure also increased astrogliosis in both sexes of *App^NL‐G‐F^* mice, as indicated by GFAP immunoreactivity. Additionally, dust‐mite exposure‐induced asthma elevated cortical levels of several cytokines in both sexes and genotypes. Finally, dust‐mite exposed groups also showed a disturbed BBB integrity in the hippocampus of *App^NL‐G‐F^* mice, as indicated by decreased collagen‐4 immunoreactivity.

**Conclusion:**

Dust‐mite exposure induced a severe asthma‐like condition that exacerbated Aβ pathology, astrogliosis, cytokine changes, and disturbed BBB integrity in the brains of male and female *App^NL‐G‐F^* mice. Defining the mechanisms of secondary effects of asthma on the brain may provide a novel therapeutic approach for both asthma and AD.

